# Overcompensation of CoA Trapping by Di(2-ethylhexyl) Phthalate (DEHP) Metabolites in Livers of Wistar Rats

**DOI:** 10.3390/ijms222413489

**Published:** 2021-12-16

**Authors:** David Hala, Lene H. Petersen, Duane B. Huggett, Michelle A. Puchowicz, Henri Brunengraber, Guo-Fang Zhang

**Affiliations:** 1 Department of Biology, University of North Texas, Denton, TX 76203, USA; petersel@tamug.edu (L.H.P.); dbh0074@gmail.com (D.B.H.); 2 Department of Marine Biology, Texas A&M at Galveston, Galveston, TX 77554, USA; 3 Boehringer Ingelheim Animal Health, Athens, GA 30601, USA; 4 Department of Nutrition, Case Western Reserve University, Cleveland, OH 44106, USA; mpuchowi@uthsc.edu (M.A.P.); henri.brunengraber@case.edu (H.B.); 5 Department of Pediatrics, The University of Tennessee Health Sciences Center, Memphis, TN 38163, USA; 6 Sarah W. Stedman Nutrition and Metabolism Center, Duke Molecular Physiology Institute, Duke University Medical Center, Durham, NC 27705, USA; guofang.zhang@duke.edu; 7 Department of Medicine, Division of Endocrinology, Metabolism Nutrition, Duke University Medical Center, Durham, NC 27710, USA

**Keywords:** DEHP, 2-ethylhexanol, CoA, CoA esters, lipid metabolism

## Abstract

Di(2-ethylhexyl) phthalate (DEHP) is commonly used as a plasticizer in various industrial and household plastic products, ensuring widespread human exposures. Its routine detection in human bio-fluids and the propensity of its monoester metabolite to activate peroxisome proliferator activated receptor-α (PPARα) and perturb lipid metabolism implicate it as a metabolic disrupter. In this study we evaluated the effects of DEHP exposure on hepatic levels of free CoA and various CoA esters, while also confirming the metabolic activation to CoA esters and partial β-oxidation of a DEHP metabolite (2-ethyhexanol). Male Wistar rats were exposed via diet to 2% (^w^/_w_) DEHP for fourteen-days, following which hepatic levels of free CoA and various CoA esters were identified using liquid chromatography-mass spectrometry. DEHP exposed rats showed significantly elevated free CoA and increased levels of physiological, DEHP-derived and unidentified CoA esters. The physiological CoA ester of malonyl-CoA and DEHP-derived CoA ester of 3-keto-2-ethylhexanoyl-CoA were the most highly elevated, at eighteen- and ninety eight-times respectively. We also detected sixteen unidentified CoA esters which may be derivative of DEHP metabolism or induction of other intermediary metabolism metabolites. Our results demonstrate that DEHP is a metabolic disrupter which affects production and sequestration of CoA, an essential cofactor of oxidative and biosynthetic reactions.

## 1. Introduction

Phthalate esters are commonly used as plasticizers in various industrial and household plastic products [1,2,3,4,5,6,7,8]. Central amongst phthalate esters is di(2-ethylhexyl) phthalate or DEHP, which along with its various metabolites, is commonly detected in the blood plasma and urine of children and adults in the general population [9,10,11,12,13,14]. Body burdens of phthalate metabolites correlate with metabolic disorders such as obesity and type-2 diabetes; and with reproductive disorders such as decreased steroid hormone levels, testicular non-descent, and premature thelarche [15,16,17,18,19].

**Figure 1:**
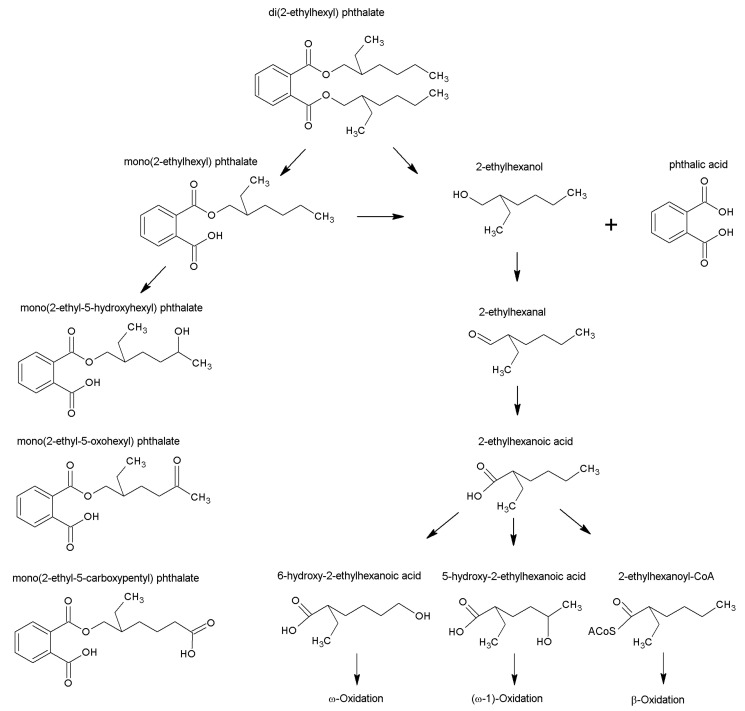
Metabolic fate of DEHP and its metabolites. Pathways are adapted from Albro [36]; Deisinger et al. [39]; Koch et al. [1]; and Rusyn et al. [40].

In rodents, DEHP acts as a metabolic disrupter and hepatic toxicant as its monoester metabolite, mono(2-ethylhexyl) phthalate (or MEHP) (Figure 1), can bind to and activate peroxisome proliferator-activated receptors (PPARs), specifically PPARα and PPARγ [20,21,22,23,24,25]. PPARs act as transcriptional ‘master regulators’ of metabolic homeostasis as they regulate genes involved with fatty acid uptake (FATP-1, Cpt1 and 2), lipid metabolism (lipoprotein lipase, acyl-CoA synthetase and acyl-CoA oxidase), and gluconeogenesis (pyruvate dehydrogenase, PDH and phosphoenolpyruvate kinase, PEPCK) [26,27]. Thus, DEHP exposure causes dysregulations of PPARα signaling and altered expression of metabolic transporter/enzyme genes with specific effects on lipid metabolism [25,28].

Any effects on lipid metabolism (i.e., fatty acid oxidation or synthesis) will involve the use of coenzyme A (or CoA) as cofactor and acyl carrier. Therefore, monitoring CoA and acyl-CoA (or CoA ester) levels is of interest as they may represent sensitive biomarkers of metabolic insult [29,30]. Furthermore, there is concern that the activation of xenobiotic carboxylic acids to xenobiotic-CoAs may cause the increased sequestration or ‘trapping’ of CoA moieties into novel (xenobiotic) acyl-CoAs, in turn depleting the cellular pool of free CoA [29,31,32]. Such CoA-trapping has been shown for several xenobiotics, such as anti-epileptic (valproate) and non-steroidal anti-inflammatory (ibuprofen) drugs [29,33,34]. In contrast to such cases of CoA-trapping, Sakurai et al. [35] have reported marked elevation of hepatic CoA and acetyl-CoA in rats treated with DEHP. Sakurai’s observation suggests that DEHP induces a global increase in liver CoA species which possibly over-compensates for any trapping of CoA in DEHP metabolites [35]. Although the formation of various DEHP-derived carboxylate metabolites has been previously predicted and reported [1,36,37,38] (Figure 1), to our knowledge none have confirmed CoA ester formation.

The goal of our study was to identify DEHP-derived CoA esters and to investigate possible perturbations in the profile of physiological CoA esters. Towards this end, we performed a 14 day dietary exposure study in which seven rats per treatment group were exposed to 20 g DEHP/Kg feed per day (or 2% (^w^/_w_) of diet) or Control diet. DEHP was shown to significantly elevate free CoA and various physiological CoA esters in hepatic tissue of exposed rats. Furthermore, we also confirmed the presence of DEHP-derived CoA conjugated 2-ethylhexanoic acid esters and detected the generation of as yet unidentified CoA esters which may be derivative of DEHP metabolism. 

## 2. Results

### 2.1. Effects of DEHP on Diet Consumption

**Figure 2:**
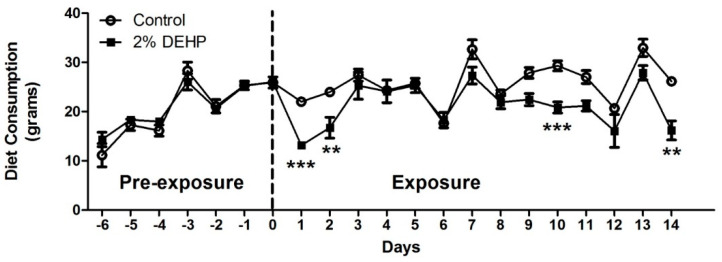
Effects of 2% DEHP exposure on diet consumption throughout the study duration (7 day pre-exposure and 14 day exposure) (*n* = 7 per group). Significant differences are indicated with; ** *p*< 0.01 and *** *p* < 0.0001.

DEHP exposure showed transient effects on diet consumption during the 14 day dietary dosing study (Figure 2). A clear and statistically significant reduction of diet consumption was evident within 48 h (Days 1 and 2) of exposure with the DEHP exposed group exhibiting a consumption rate 56% that of the previous or pre-exposure day. Subsequently, significantly decreased consumption was also observed for Days 10 and 14 (two-way ANOVA with Bonferroni post-test, *p* < 0.05). 

### 2.2. Effects of DEHP on Rodent Growth and Liver Weight

**Figure 3:**
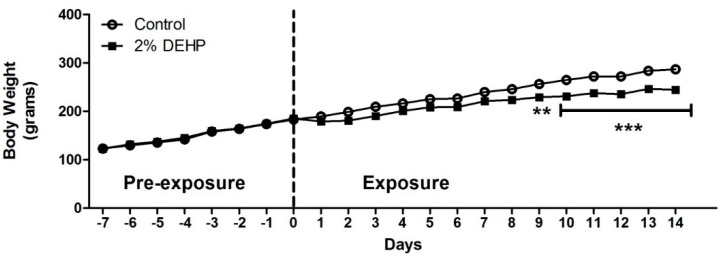
Effects of 2% DEHP exposure on body weights of male Wistar rats throughout the study duration (7 day pre-exposure and 14 day exposure) (*n* = 7 per group). Significant differences are indicated with; ** *p* < 0.01 and *** *p* < 0.0001.

**Figure 4:**
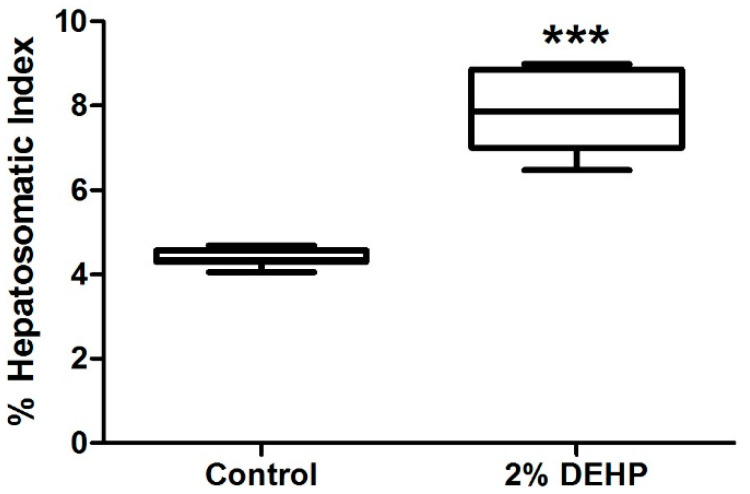
Effect of 2% DEHP exposure on hepatosomatic index of male Wistar rats as quantified on termination of the 14 day exposure study. Significant difference is indicated with; *** = *p* < 0.001.

A significant decrease of rat body weights was evident from Day 9 and onwards for the 2% DEHP exposed group (*p* < 0.05) (Figure 3). In contrast, hepatosomatic index showed a significant increase in liver weights for 2% DEHP exposed rats relative to Control (Figure 4). While body weight loss is likely due to reduced diet consumption and elevated lipid metabolism (contributing to a ‘lean’ phenotype) [25,35]; increased liver weight suggests hepatomegaly due to peroxisomal proliferation [41,42].

### 2.3. Effects of DEHP on CoA Ester Production

**Figure 5:**
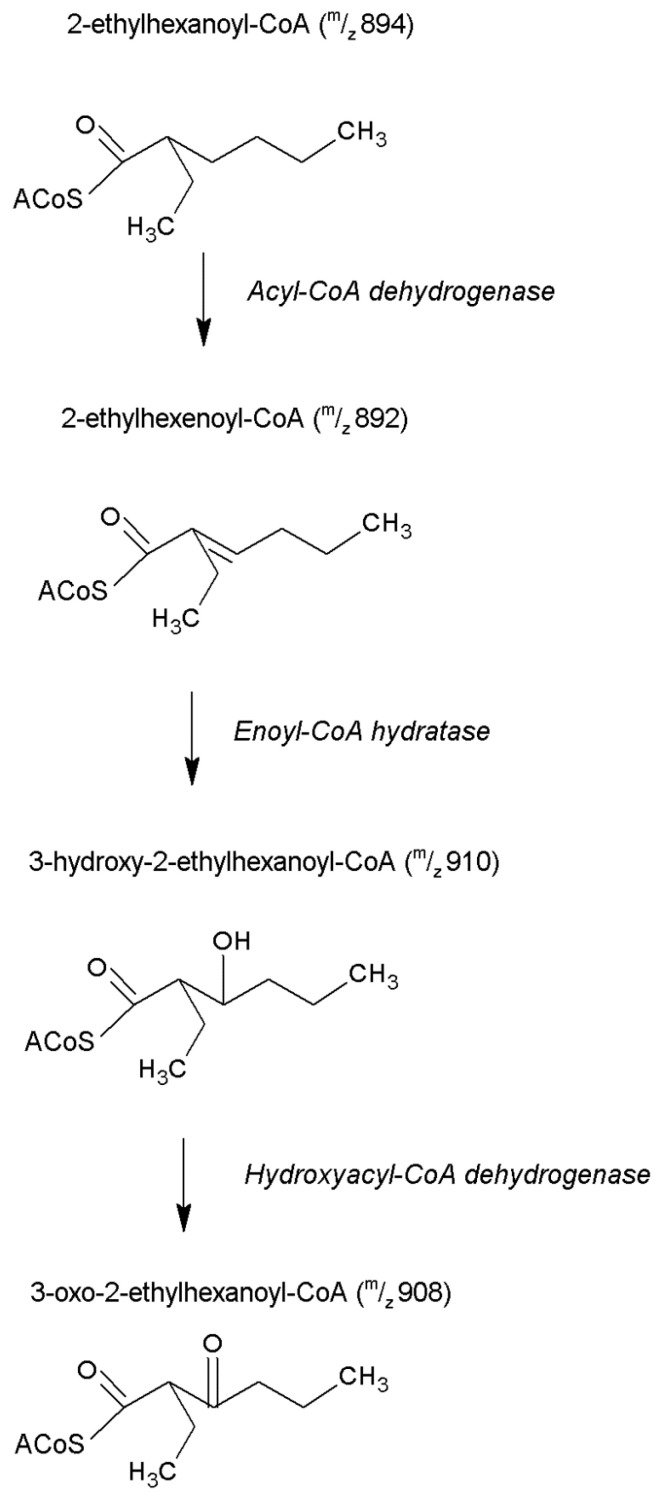
Metabolic pathway detailing the partial β-oxidation of 2-ethylhexanoyl-CoA to 3-oxo-2-ethylhexanoyl-CoA (adapted from Walker and Mills [37]; English et al. [43]). The mass transitions listed for each intermediate metabolite (in parenthesis) were confirmed by mass spectrometry in our study.

Exposure to 2% DEHP induced a significant increase (10-times) in free CoA concentration relative to Control (Table 1). Overall, the total pool of CoA (free + esterified) was increased by DEHP exposure. The DEHP-derived CoA esters listed in the second section of Table 1 are isobars of *n*-C_8_ intermediates of physiological β-oxidation (as also identified in Control livers). The most abundant of the CoA esters derived from DEHP is likely 2-ethylhexanoyl-CoA, which is an isobar of, and which has the same HPLC retention time as octanoyl-CoA. It is thus very likely that the C_8_ CoA esters detected in livers from DEHP-treated rats contain a small component of physiological *n*-C_8_ CoA esters. Overall, the presence of 2-ethylhexanoyl-CoA, 2-ethyl-2-hexenoyl-CoA, 3-hydroxy-2-ethylhexanoyl-CoA and 3-keto-2-ethylhexanoyl-CoA strongly indicates partial β-oxidation of CoA conjugated 2-ethylhexanoic acid (Figure 5). Finally, increased levels for various unidentified CoA esters were also observed (last section of Table 1). 

## 3. Discussion

### 3.1. DEHP Feeding Study 

Our observation of reduced body weight agreed with other published studies [25,35]. The hepatosomatic index showed a significant increase in liver weights for 2% DEHP dosed rats relative to Control. Such hepatomegaly has been reported previously [25,35], and is ascribed to induction of peroxisomal proliferation [31,44]. The levels of DEHP used in our study far exceed those (by four orders of magnitude) detected in human bio-fluids. For example, maximal estimated daily dietary consumption rate for DEHP in adult humans can range from 0.7 to 36.5 µg DEHP/kg/day [3,45,46]. While maximal levels (95th percentile) of 52 and 1780 µg DEHP/kg/day have been calculated for adults and premature neonates receiving intensive medical care (due to high exposure to plastic products) [1,10]. Given a sub-chronic/chronic no observed adverse effect level (NOAEL) for endocrine effects of >100 mg DEHP/Kg/day from rodent toxicity studies [47]; the highest level of ~2 mg DEHP/kg/day in neonates and a tolerable daily intake (TDI) rate for humans of 37 mg DEHP/Kg/day [48] may temper concerns of DEHP’s metabolic disruptive potential in humans. However, the detection of partial β-oxidation metabolites of 2-ethylhexanoic acid in urine from patients receiving intravenous infusions (children born with congenital metabolic disorders of medium-chain acyl-CoA dehydrogenase (MCAD) deficiency) confirms biotransformation of a DEHP metabolite in CoA participating pathways (Figure 1) [37]. Hence, DEHP may act as a metabolic disrupter by modulating CoA and acyl-CoA metabolisms. The exhibition of such propensity in our feeding study is described further below.

### 3.2. Physiological CoA Esters

The first section of Table 1 reports on physiological CoA esters which are unequivocally identified in the livers of Control and of DHEP exposed rats. Because labeled internal standards are not available for these compounds, concentrations are expressed relative to an internal standard of [^2^H_9_]pentanoyl-CoA. However, the ratio of relative concentrations (DEHP)/(Control), equals the ratio of absolute concentrations of (DEHP)/(Control) because of the cancelation of the internal standard in the calculation. For the same reason, within one group (such as within Control or DEHP exposed), one can calculate valid metabolite ratios such as [acetyl-CoA]/[CoA]. 

Our results show that exposure to 2% DEHP induced a significant (10-times) increase in free CoA concentration relative to Control. This leaves open to investigation the mechanisms fueling increase of free CoA while concomitantly elevating physiological acyl-CoA productions under DEHP exposure. Tissue CoA levels are maintained by balancing between synthesis, degradation, and renewal pathways. CoA synthesis is under the control of pantothenate kinase (or Pank) which catalyzes the first rate-limiting step in CoA synthesis (i.e., catalysis of pantothenic acid to 4′-phosphopantothenic acid) [49]. The human Pank gene (hPank1α) bares a PPARα response element in its promoter region, associating the ability of hypolipidemic agents (such as bezafibrate) to induce CoA synthesis [50]. Skrede and Halvorsen [51] show elevated biosynthesis of CoA in rats exposed to the peroxisome proliferator, clofibrate. The authors demonstrated elevated (up to 4-times relative to Control) ^14^C label incorporation from [^14^C]pantothenate to CoA [51]. Furthermore, PPARα activation has also been shown to down-regulate the expression of CoA degrading enzymes such as nudix (NUDT) hydrolases (specifically NUDT7α) [52]; further lending support to the association of PPAR modulation by DEHP (and its mono-ester metabolite, MEHP) and perturbed CoA homeostasis. More recently, the induction of a CoA salvage (or renewal) pathway catalyzed by pantetheinases (belonging to the Vanin protein family) have also been evoked as being capable of hydrolyzing pantetheine (produced from 4′-phosphopantetheine in the CoA biosynthesis pathway) to pantothenic acid, which in turn is reused for CoA synthesis [53]. In addition, Vanin gene expression is under regulatory control by PPARα [54]; and has been shown to be induced under DEHP exposure [28]. 

An additional mechanism supporting CoA generation can involve the elevated degradation of high acetyl-CoA levels. Leighton et al. [55] showed hepatocytes isolated from bezafibrate treated rats (and incubated with [^14^C]dodecanedioic acid) to produce elevated levels of [^14^C]acetate (with minimal amounts of β-hydroxybutyrate and acetoacetate). The authors suggest that most of the acetyl-CoA generated by enhanced fatty acid β-oxidation was converted to acetate via the activity of cytosolic acetyl-CoA hydrolases. The release of free acetate can constitute a mechanism via which CoA is recycled, therefore also contributing to the prevention of CoA trapping. Finally, Himms-Hagen and Harper [56] propose that peroxisome proliferators can induce the activity of mitochondrial acyl-CoA thioesterases that convert mitochondrial acyl-CoAs to fatty acid anions. These fatty acid anions can be exported out of the mitochondria by UCP3 (or un-coupling protein 3 anion transporter), only to be shuttled back into the mitochondria by the enhanced activity acyl-CoA synthetases (due to PPARα mediated induction of β-oxidation). This in effect creates a ‘futile-cycle’ liberating CoA during high demand for β-oxidation pathways, while preventing CoA trapping. It is unclear whether some (or all) of these mechanisms are responsible for our observation of increased free CoA in DEHP exposed rats, however they provide plausible avenues for further investigation.

In our study, the comprehensive analysis of hepatic CoA-thioesters showed overall increases for various acyl-CoAs in 2% DEHP exposed rats relative to Control (Table 1). Such elevated acyl-CoA levels indicate enhanced activation of fatty acids to CoA thioesters and elevated lipid metabolism. The observed increase of acetyl-CoA (2-times) relative to the Control group is symptomatic of two-carbon decarboxylation’s of fatty acyl-CoAs in fatty acid β-oxidation, with increase of short-chain (C2–C5) fatty acyl-CoAs indicating mainly mitochondrial β-oxidation [41]. Our data also suggests ‘overflow’ metabolism of elevated acetyl-CoA to succinyl-CoA (via TCA cycle) and malonyl-CoA (which can fuel fatty acid synthesis) [57,58]. Interestingly, malonyl-CoA was the most highly induced in DEHP exposed rats (18-times) relative to Control. Elevated acetyl-CoA and malonyl-CoA can be expected to have wide-reaching metabolic effects. For example, acetyl-CoA can perturb the TCA cycle by inhibiting pyruvate dehydrogenase (PDH) activity and preventing pyruvate entry into the TCA cycle [59]. Elevated malonyl-CoA can suppress mitochondrial uptake of fatty acids (via inhibition of carnitine palmitoyl transferase-1) and in turn perturb fatty acid β-oxidation [58,60]. The data as presented cannot reconcile the extent to which underlying metabolic flux distributions between lipid (fatty acid oxidation and synthesis) and carbohydrate metabolisms are perturbed. However, this lends to further investigation using stably labeled isotopic tracers.

Elevated propionyl-CoA levels in 2% DEHP exposed rats (2-times that of Control) indicate β-oxidation of odd chain fatty acids (such as pentadecanoic (C15) acid), which can generate large amounts of both propionyl-CoA and acetyl-CoA [61]. Propionyl-CoA can be carboxylated (via propionyl-CoA carboxylase) to produce S-methylmalonyl-CoA and R-methymalonyl-CoA (via methylmalonyl-CoA racemase). These in turn are converted to succinyl-CoA by the activity of methylmalonyl-CoA mutase, thus potentially contributing to the large amounts of succinyl-CoA observed in our study (3-times that of Control) [62]. The high levels of propionyl-CoA (2-times relative to Control), methylmalonyl-CoA (3-times) and succinyl-CoA (3-times) support this premise, especially as perfusion of rat heart and liver with [^13^C_3_]-labelled propionate shows isotopic enrichment of propionyl-CoA, methylmalonyl-CoA and succinyl-CoA [63,64]. The possibility of both acetyl-CoA and methylmalonyl-CoA ‘draining-into’ succinyl-CoA production can maintain TCA cycle function and generation of reducing equivalents (such as NADH) for ATP production (via electron transport chain). Support for this hypothesis is provided by Sakurai et al.’s [35] observation of no differences in hepatic pools of nucleotide phosphates (ATP, ADP and AMP) in livers of Wistar rats exposed to 2% DEHP. Furthermore, calculation of energy charge for the control (0.72) and 2% DEHP exposed rats (0.74) also confirmed no effects on ATP production under DEHP exposure.

### 3.3. DEHP-Derived CoA Esters Which Are Isobars of Physiological CoA Esters

Previous studies have identified a number of carboxylic acids derived from the 2-ethylhexanol moiety of DEHP [36,37]. 2-Ethylhexanol is oxidized in the liver to 2-ethylhexanoate via alcohol and aldehyde dehydrogenases [36] and via the catalase-H_2_O_2_ system [65]. Further metabolism of 2-ethylhexanoate can involve β-oxidation as well as ω- and ω-1 oxidation (Figure 1) [37,39]. We could therefore make reasonable predictions on the ^m^/_z_ of CoA esters derived from C_8_ 2-ethylhexanoate. These DEHP-derived CoA esters are listed in the second section of Table 1. Note that these esters are isobars of *n*-C_8_ intermediates of physiological β-oxidation, identified in Control livers. The most abundant of the CoA esters derived from DEHP is likely 2-ethylhexanoyl-CoA, which is an isobar of (and with the same HPLC retention time) as octanoyl-CoA. It is thus very likely that the C_8_ CoA esters detected in livers from DEHP-treated rats contain a small component of physiological *n*-C_8_ CoA esters. 

In DEHP-exposed livers, the very high relative concentration of 2-ethylhexanoyl-CoA (37-times that of octanoyl-CoA in Control) reflects the very slow metabolism of this compound. This conclusion is supported by the concentrations of C_6_ and C_4_ CoA esters (hexanoyl-CoA and butyryl-CoA) which are similar to Control values. The identification of 2-ethyl-2-hexenoate, 3-hydroxy-2-ethyl-hexanoate, and 3-keto-2-ethylhexanoate CoA esters in DEHP-treated rats reflects the sequence 2-ethylhexanoyl-CoA→2-ethyl-2-hexenoyl-Co→3-hydroxy-2-ethylhexanoyl-Co→3-keto-2-ethylhexanoyl-CoA (Figure 5). The thiolytic cleavage of 3-keto-2-ethylhexanoyl-CoA to two butyryl-CoA molecules by 3-ketoacyl-CoA thiolase may be very slow. Thus, 2-ethylhexyl-CoA may be disposed by (i) hydrolysis to the free acid which is excreted in urine [37], and/or (ii) by conversion to a carnitine ester in view of the reported activation of carnitine acyltransferase by ethylhexanoate [66].

### 3.4. Unidentified CoA Esters

As seen in the last section of Table 1, increased levels for various unidentified CoA esters was surprising and indicated widespread sequestration of CoA into either DEHP derived or intermediary metabolism metabolites. The detection of the CoA ester of ^m^/_z_ 904 (Unknown 6) suggests the detection of a C_n-4_ 3-keto-2-ethylhexanoyl-CoA metabolite having undergone two successive 2-carbon unit removals; however this is unverifiable at present. It is possible that steric hindrance from the ethyl group on carbon-2 position of 3-keto-2-ethylhexanoyl-CoA hinders 3-ketoacyl-CoA thiolases catalytic activity, or that this final β-oxidation enzyme’s activity is inhibited by the increasing acetyl-CoA levels observed under DEHP exposure [67]. This speculation is supported as the unidentified CoA ester ‘Unknown 6′ also exhibited the highest induction of production, at 190-times that of levels in the Control group. Furthermore, its putative precursor 3-keto-2-ethylhexanoyl-CoA was the second most highly induced DEHP-derived CoA ester (at 98-times relative to Control). 

### 3.5. Constraints on the LC-MS/MS Analyses of CoA Esters

The following constraints affect the mass spectrum analysis of all CoA esters. First, fragmentations of the molecular ion occur only in the CoA moiety (^m^/_z_ 767), without affecting the carboxylate moiety [68,69]. The mass of the carboxylate moiety can be easily calculated as M-767. The initial fragmentation of a CoA ester ion of mass M yields (i) a M-507 ion that includes the carboxylate group, and (ii) a m/z 428 ion, formed from all CoA esters, which includes adenine + ribose + 2 phosphate groups [68]. Further fragmentations of the M-507 ion yield smaller ions that still contain the intact carboxylate group. Therefore, if two or more CoA esters with the same mass (isobars) have the same retention time on the HPLC column, they cannot be distinguished from the LC-MS/MS data. This feature impacts the interpretation of our data on CoA esters analysis. Second, few authentic standards of CoA esters (unlabeled and stable-isotope-labeled) are commercially available; therefore, identification of unknown CoA esters is based on the above transitions. Third, when unlabeled and labeled standards of CoA esters are available, the slopes of the calibration curves can markedly vary. Thus, when using one or few labeled internal standards, one can only calculate relative concentrations of CoA esters. These relative concentrations are similar to those generated in most metabolomic studies. Still, these relative concentrations allow calculating relative concentrations of given CoA esters between groups. 

## 4. Materials and Methods

### 4.1. Materials

DEHP and deuterated [3,4,5,6-d_4_]bis(2-ehylhexyl) phthalate ([d_4_]-DEHP) internal standard were purchased from Alfa Aesar and Sigma, respectively. Dichloromethane (DCM) was used as diluent for all standard preparations which were stored at −20 °C. Because of the high likelihood of phthalate contamination from plastics, only glass syringes with stainless steel (or glass) plungers were used to prepare stocks and aliquot materials.

### 4.2. DEHP Diet Preparation

Rodent diet was purchased as ground meal from Harlan Laboratories (Teklad Rodent Diet, Cat.# T.8604M.15). The powder was mixed with a 2% emulsion of DEHP in water (prepared by sonication). Control diet was mixed with water and the pasty diet was dried in an oven (overnight) at 90 °C. 

### 4.3. GCMS Quantification of DEHP in Diet

A Hewlett Packard 5890 series II gas chromatograph with electron impact ionization (EI) was used for analyte quantification. An Agilent DB-5 column was used for capillary separations (30m × 0.25 mm × 0.25 µm film thickness). Oven temperature-ramping began from a set-point of 40 °C (initial hold of 3 min); ramped at 25 °C/min to 190 °C; then ramped at 30 °C/min to 300 °C; and held for an 8 min bake-out (total runtime of 20.67 min). Solvent delay was up to 13 min with the detector turned off at 16 min. DEHP and d_4_-DEHP retention times were at approximately 14 min. Detector and injector temperature was set to 250 °C respectively. Helium was used as carrier gas with a 21 psi head pressure yielding ~1.1 mL/min column flow. Samples were injected (1 µL volume) with a split ratio of 100:1. The mass detector collected data in SIM mode (dwell time of 50 milliseconds for each ion). Quantifying and confirming ions were as follows: DEHP = 149 (quantifying ion), 167 and 279 (confirming ions); and for [d_4_]-DEHP = 153 (quantifying ion), 171 and 283 (confirming ions). Dosing optimization studies conducted prior to initiation of the 14 day dosing study gave actual concentrations 107% of the intended nominal of 2% DEHP (or 20 g DEHP/Kg diet), this equated to a concentration of 21.43 g DEHP/Kg diet (±1.37 standard error of mean or s.e.m). 

### 4.4. In Vivo Experiment

All animal handling procedures were approved by the University of North Texas IACUC. Fourteen male Wistar rats (32–35 days; 123 ± 4 g) were purchased from Charles River Laboratories. On arrival, all rats were housed in individual cages in a temperature controlled room (70–80 °F) with a 12 h light:12 h dark cycle. Food consumption and body weight were measured daily throughout the study. All rats were maintained for a 7 day pre-exposure period to allow acclimation. During this time all rats were fed the Control (un-tainted) diet *ad libitum*. Then, seven rats were exposed for 14 days to a diet containing 2% DEHP by weight of feed. The remainder 7 rats were kept on the Control diet. Food and water was available *ad libitum*. On termination of the 14 day exposure study, rats were anesthetized with 2% isoflurane, and the liver was dissected out and weighed. Then a section of the large upper lobe of the liver was clamp frozen and immediately stored in liquid nitrogen for analysis of tissue levels of free CoA and CoA esters.

### 4.5. LC-MS/MS Quantification of Free CoA and CoA Esters

Free CoA and CoA esters were analyzed by LC-MS/MS (4000 QTRAP, Applied Biosystems, Foster City, CA) using a previously published method [70]. Briefly, liver samples were powdered in liquid nitrogen, and 200 mg powdered tissues were weighted for CoA and CoA ester assay. [2,2,3,3,3-^2^H_5_]propionyl-CoA (0.2 nmol) and [2,2,3,3,4,4,5,5,5-^2^H_9_ ]pentanoyl-CoA (0.2 nmol) were used as internal standards. CoA and CoA esters were extracted in 5% acetic acid water/methanol (1:1, v/v) solution and purified by weak anion cartridge [69]. The eluent from cartridge was dried by N_2_ gas and made ready for LC-MS/MS analysis after the dried residue was suspended in 100 µL Milli-Q water. Detection of free CoA and CoA esters was based on the M-507 precursor/product transition [68].

### 4.6. Statistical Analysis

Statistical analyses were performed using GraphPad Prism^TM^ version 5 (GraphPad Software, La Jolla, CA). All datasets were tested for Normality using the Shapiro–Wilk’s test (normal distribution at *p* ≥ 0.05). The hepatosomatic index, body weight and hepatic levels of free CoA and CoA esters were statistically compared using either the parametric unpaired t-test (if normal distribution) or non-parametric Mann–Whitney test (non-normal distribution). For datasets in which the Control level of a CoA ester was non-detectable, the non-parametric Wilcoxon signed rank test was used to compare median value of the DEHP exposed group versus a hypothetical (or null) median value representing the Control group. Two-way analysis of variance (ANOVA) was used to test for effects of DEHP exposure on diet consumption and rat body weight during the entire course of the study (i.e., inclusive of 7 day pre-exposure and 14 day exposure). For testing with two-way ANOVA the 2% DEHP exposure dose was used as the main effects variable and time (or duration of study) used as a covariate. In all cases significance was declared at *p* < 0.05. 

## 5. Conclusions

A key finding of our study was that DEHP exposure greatly increased hepatic free CoA and CoA ester levels. The elevation of free CoA, despite high sequestration into CoA esters, demonstrates overcompensation of CoA trapping in DEHP-derived or intermediary fatty acyl-CoAs. As CoA constitutes a key cofactor participant in various oxidative and biosynthetic reactions, the altered CoA/acyl-CoA ratios reported in our study open new avenues for investigating DEHPs endocrine and metabolic disrupting potential. Furthermore, the overcompensation of CoA trapping contrasts with what occurs in some inborn errors of metabolism. For example, hereditary disorders of impaired lipid metabolism show accumulation of acyl-CoAs concomitant with depleted CoA [71,72]. The increased sequestration and redistribution of free CoA (into acyl-CoAs) contributes to its depletion, causing metabolic stress whose etiologies are encompassed by the CASTOR (Coenzyme A sequestration, toxicity and redistribution) phenomena [73]. The overcompensation of CoA trapping most likely involves a major activation of the pantothenate kinase pathway [74]. Identifying the mechanisms driving elevated CoA synthesis may help in the treatment of CASTOR conditions. Finally, we also confirmed that the DEHP metabolite, 2-ethyhexanol, is converted to a CoA ester and (at least) partially metabolized in fatty acid β-oxidation. The further identification of DEHP-derived CoA esters will require complementary experiments with DEHP isotopically labeled in the phthalate and 2-ethylhexanol moiety.

## Figures and Tables

**Table 1:** Relative concentrations of free CoA and CoA esters identified by the M-507 transition during LC-MS/MS analysis of livers from control and DEHP exposed rats (*n* = 7 per group) (nmol/g or nmol/g tissue). Because labeled internal standards are not available for most analytes, the data were calculated as relative concentrations compared to an internal standard of [^2^H_9_]pentanoyl-CoA means ± s.e.m. Thus, relative concentrations of different CoA esters may not be added to estimate the total pool of CoA. However, in the 1st section of the table (free CoA and physiological CoA esters), the ratios of relative concentrations [DEHP]/[Control] in the last column represent valid variations in the liver content of the corresponding analyte. The 2nd section of the table lists four C_8_ physiological CoA esters and some DEHP-derived CoA esters which are isobars with the same retention times as the four C_8_ physiological CoA esters. In this section, the ^m^/_z_ 892 and 910 could be any of two or three DEHP-derived CoA esters, respectively. The 3rd section of the table lists unidentified CoA esters in Control livers which are isobars with the same retention times as unidentified CoA esters in livers from DEHP-treated rats. Note that in the 2nd and 3rd section of the table, CoA esters listed under 2% DEHP probably include CoA esters listed under Control. Thus, the high [DEHP]/[Control] ratios in the last column reflect the likely formation of unknown DEHP-derived CoA esters. *, **, *** denote significant difference relative to Control at: *p* < 0.05, *p* ≤ 0.01, *p* ≤ 0.001, respectively.

	Retention Time (min)	Control nmol/g	2% DEHP nmol/g	[DEHP]/[Control] ^1^
*Free CoA and physiological CoA esters*				
^m^/_z_ 768: Free CoA	4.6	36.1 ± 3.1	364.3 ± 64.5 ***	10
^m^/_z_ 810: Acetyl-CoA	8.1	97.7 ± 4.0	190.6 ± 18.0 ***	2
^m^/_z_ 824: Propionyl-CoA	8.9	7.9 ± 1.1	18.0 ± 1.7 ***	2
^m^/_z_ 838: Butyryl-CoA	9.6	19.8 ± 4.0	29.2 ± 2.4	1.5
^m^/_z_ 852: Pentanoyl-CoA	10.3	7.7 ± 0.7	11.0 ± 0.4 **	1.4
^m^/_z_ 854: Malonyl-CoA	3.2	0.2 ± 0.03	2.8 ± 0.7 **	18
^m^/_z_ 854: β-hydroxybutyryl-CoA	8.3	9.0 ± 1.3	9.1 ± 1.1	1.0
^m^/_z_ 866: Hexanoyl-CoA	11.0	12.0 ± 3.1	13.1 ± 1.8	1.1
^m^/_z_ 868: Succinyl-CoA	6.1	3.3 ± 0.5	9.2 ± 1.4 **	3
^m^/_z_ 868: Methylmalonyl-CoA	5.1	0.4 ± 0.1	1.1 ± 0.2 *	3
^m^/_z_ 880: Heptanoyl-CoA	11.5	3.4 ± 0.3	4.0 ± 0.3	1.2
^m^/_z_ 912: 3-Hydroxy-3-methylglutaryl-CoA	7.3	5.0 ± 0.7	3.2 ± 0.6	0.6
^m^/_z_ 922: Decanoyl-CoA	12.8	0.5 ± 0.1	0.3 ± 0.04	0.6
^m^/_z_ 1006: Palmitoyl-CoA	14.7	2.2 ± 0.4	3.4 ± 1.0	2
*Identified DEHP-derived CoA esters which are isobars of physiological CoA esters*				
^m^/_z_ 892: 3-Octenoyl-CoA 2-Ethyl-2-hexenoyl-CoA 2-Ethyl-5-hexenoyl-CoA	11.5	4.5 ± 0.4	53.4 ± 8.8 ***	12
^m^/_z_ 894: Octanoyl-CoA 2-Ethylhexanoyl-CoA	11.6	11.8 ± 2.6	438.7 ± 59.1 ***	37
^m^/_z_ 908: 3-Ketooctanoyl-CoA 3-Keto-2-ethylhexanoyl-CoA	10.7	0.1 ± 0.1	12.5 ± 1.7 **	98
^m^/_z_ 910: 3-Hydroxyoctanoyl-CoA 3-Hydroxy-2-ethylhexanoyl-CoA 6-Hydroxy-2-ethylhexanoyl-CoA 5-Hydroxy-2-ethylhexanoyl-CoA	10.3	1.0 ± 0.1	23.2 ± 3.9 **	22
*Unidentified CoA esters*				
^m^/_z_ 861: Unknown 1	9.4	0.1 ± 0.02	2.0 ± 0.3 ***	14
^m^/_z_ 862: Unknown 2	7.9	0.2 ± 0.03	0.7 ± 0.1 ***	4
^m^/_z_ 872: Unknown 3	10.2	0.1 ± 0.03	1.5 ± 0.2 ***	14
^m^/_z_ 875: Unknown 4	9.9	0.2 ± 0.03	2.9 ± 0.5 ***	16
^m^/_z_ 888: Unknown 5	9.7	0.6 ± 0.1	2.6 ± 0.6 ***	4
^m^/_z_ 904: Unknown 6	9.0	0.04 ± 0.04	7.1 ± 1.6 **	190
^m^/_z_ 929: Unknown 7	9.3	0.02 ± 0.01	0.5 ± 0.1 **	23
^m^/_z_ 947: Unknown 8	10.4	0.02 ± 0.01	0.6 ± 0.1 **	32
^m^/_z_ 968: Unknown 9	9.5	ND	0.4 ± 0.1 *	-
^m^/_z_ 989: Unknown 10	10.9	0.01 ± 0.01	0.1 ± 0.1	17
^m^/_z_ 996: Unknown 11	11.8	ND	0.2 ± 0.1 *	-
^m^/_z_ 998: Unknown 12	11.9	0.03 ± 0.02	0.3 ± 0.1 **	11
^m^/_z_ 1008: Unknown 13	10.4	ND	0.4 ± 0.1 *	-
^m^/_z_ 1026: Unknown 14	8.2	ND	0.5 ± 0.1 *	-
^m^/_z_ 1030: Unknown 15	9.8	0.1 ± 0.1	9.6 ± 1.6 ***	80
^m^/_z_ 1052: Unknown 16	10.5	ND	0.3 ± 0.1 *	-

^1^ Ratio of [DEHP]/[Control] with values <2 are shown to 1 decimal place; ND = no CoA ester detected at that ^m^/_z_ and retention time.

## References

[1] Koch H.M., Preuss R., Angerer J. (2006). Di(2-ethylhexyl)phthalate (DEHP): Human metabolism and internal exposure—An update and latest results. Int. J. Androl..

[2] Hatch E.E., Nelson J.W., Stahlhut R.W., Webster T.F. (2010). Association of endocrine disruptors and obesity: Perspectives from epidemiological studies. Int. J. Androl..

[3] Schecter A., Lorber M., Guo Y., Wu Q., Yun S.H., Kannan K., Hommel M., Imran N., Hynan L.S., Cheng D. (2013). Phthalate concentrations and dietary exposure from food purchased in New York State. Environ. Health Perspect..

[4] Dewalque L., Charlier C., Pirard C. (2014). Estimated daily intake and cumulative risk assessment of phthalate diesters in a Belgian general population. Toxicol. Lett..

[5] Kim S.H., Park M.J. (2014). Phthalate exposure and childhood obesity. Ann. Pediatr. Endocrinol. Metab..

[6] Zhang Y., Meng X., Chen L., Li D., Zhao L., Zhao Y., Li L., Shi H. (2014). Age and sex-specific relationships between phthalate exposures and obesity in Chinese children at puberty. PLoS ONE.

[7] Grindler N.M., Allsworth J.E., Macones G.A., Kannan K., Roehl K.A., Cooper A.R. (2015). Persistent organic pollutants and early menopause in U.S. women. PLoS ONE.

[8] Calafat A.M., McKee R.H. (2006). Integrating biomonitoring exposure data into the risk assessment process: Phthalates [diethyl phthalate and di(2-ethylhexyl) phthalate] as a case study. Environ. Health Perspect..

[9] Blount B.C., Silva M.J., Caudill S.P., Needham L.L., Pirkle J.L., Sampson E.J., Lucier G.W., Jackson R.J., Brock J.W. (2000). Levels of seven urinary phthalate metabolites in a human reference population. Environ. Health Perspect..

[10] Koch H.M., Drexler H., Angerer J. (2003). An estimation of the daily intake of di(2-ethylhexyl)phthalate (DEHP) and other phthalates in the general population. Int. J. Hyg. Environ. Health.

[11] Silva M.J., Samandar E., Preau J.L., Needham L.L., Calafat A.M. (2006). Urinary oxidative metabolites of di(2-ethylhexyl) phthalate in humans. Toxicology.

[12] Guo Y., Wu Q., Kannan K. (2011). Phthalate metabolites in urine from China, and implications for human exposures. Environ. Int..

[13] Goen T., Dobler L., Koschorreck J., Muller J., Wiesmuller G.A., Drexler H., Kolossa-Gehring M. (2011). Trends of the internal phthalate exposure of young adults in Germany—Follow-up of a retrospective human biomonitoring study. Int. J. Hyg. Environ. Health.

[14] Song N.R., On J.W., Lee J., Park J.D., Kwon H.J., Yoon H.J., Pyo H. (2013). Biomonitoring of urinary di(2-ethylhexyl) phthalate metabolites of mother and child pairs in South Korea. Environ. Int..

[15] Colon I., Caro D., Bourdony C.J., Rosario O. (2000). Identification of phthalate esters in the serum of young Puerto Rican girls with premature breast development. Environ. Health Perspect..

[16] Swan S.H. (2008). Environmental phthalate exposure in relation to reproductive outcomes and other health endpoints in humans. Environ. Res..

[17] Pan G., Hanaoka T., Yoshimura M., Zhang S., Wang P., Tsukino H., Inoue K., Nakazawa H., Tsugane S., Takahashi K. (2006). Decreased serum free testosterone in workers exposed to high levels of di-n-butyl phthalate (DBP) and di-2-ethylhexyl phthalate (DEHP): A cross-sectional study in China. Environ. Health Perspect..

[18] Joensen U.N., Frederiksen H., Blomberg J.M., Lauritsen M.P., Olesen I.A., Lassen T.H., Andersson A.M., Jorgensen N. (2012). Phthalate excretion pattern and testicular function: A study of 881 healthy Danish men. Environ. Health Perspect..

[19] Lind P.M., Zethelius B., Lind L. (2012). Circulating levels of phthalate metabolites are associated with prevalent diabetes in the elderly. Diabetes Care.

[20] Issemann I., Green S. (1990). Activation of a member of the steroid hormone receptor superfamily by peroxisome proliferators. Nature.

[21] Gottlicher M., Widmark E., Li Q., Gustafsson J.A. (1992). Fatty acids activate a chimera of the clofibric acid-activated receptor and the glucocorticoid receptor. Proc. Natl. Acad. Sci. USA.

[22] Issemann I., Prince R.A., Tugwood J.D., Green S. (1993). The peroxisome proliferator-activated receptor: Retinoid X receptor heterodimer is activated by fatty acids and fibrate hypolipidaemic drugs. J. Mol. Endocrinol..

[23] Roberts R.A. (1999). Peroxisome proliferators: Mechanisms of adverse effects in rodents and molecular basis for species differences. Arch. Toxicol..

[24] Feige J.N., Gelman L., Rossi D., Zoete V., Metivier R., Tudor C., Anghel S.I., Grosdidier A., Lathion C., Engelborghs Y. (2007). The endocrine disruptor monoethyl-hexyl-phthalate is a selective peroxisome proliferator-activated receptor gamma modulator that promotes adipogenesis. J. Biol. Chem..

[25] Feige J.N., Gerber A., Casals-Casas C., Yang Q., Winkler C., Bedu E., Bueno M., Gelman L., Auwerx J., Gonzalez F.J. (2010). The pollutant diethylhexyl phthalate regulates hepatic energy metabolism via species-specific PPARalpha-dependent mechanisms. Environ. Health Perspect..

[26] Berger J., Moller D.E. (2002). The mechanisms of action of PPARs. Annu. Rev. Med..

[27] Rakhshandehroo M., Knoch B., Muller M., Kersten S. (2010). Peroxisome proliferator-activated receptor alpha target genes. PPAR Res..

[28] Eveillard A., Lasserre F., de Tayrac M., Polizzi A., Claus S., Canlet C., Mselli-Lakhal L., Gotardi G., Paris A., Guillou H. (2009). Identification of potential mechanisms of toxicity after di-(2-ethylhexyl)-phthalate (DEHP) adult exposure in the liver using a systems biology approach. Toxicol. Appl. Pharmacol..

[29] Knights K.M. (1998). Role of hepatic fatty acid: Coenzyme A ligases in the metabolism of xenobiotic carboxylic acids. Clin. Exp. Pharmacol. Physiol..

[30] Martinez D.L., Tsuchiya Y., Gout I. (2014). Coenzyme A biosynthetic machinery in mammalian cells. Biochem. Soc. Trans..

[31] Lock E.A., Mitchell A.M., Elcombe C.R. (1989). Biochemical mechanisms of induction of hepatic peroxisome proliferation. Annu. Rev. Pharmacol. Toxicol..

[32] Brass E.P. (1994). Overview of coenzyme A metabolism and its role in cellular toxicity. Chem. Biol. Interact..

[33] Knights K.M., Drew R. (1992). The effects of ibuprofen enantiomers on hepatocyte intermediary metabolism and mitochondrial respiration. Biochem. Pharmacol..

[34] Silva M.F., Aires C.C., Luis P.B., Ruiter J.P., IJlst L., Duran M., Wanders R.J., Tavares de Almeida I. (2008). Valproic acid metabolism and its effects on mitochondrial fatty acid oxidation: A review. J. Inherit. Metab. Dis..

[35] Sakurai T., Miyazawa S., Hashimoto T. (1978). Effects of di-(2-ethylhexyl)phthalate administration on carbohydrate and fatty acid metabolism in rat liver. J. Biochem..

[36] Albro P.W. (1975). The metabolism of 2-ethylhexanol in rats. Xenobiotica.

[37] Walker V., Mills G.A. (2001). Urine 4-heptanone: A beta-oxidation product of 2-ethylhexanoic acid from plasticisers. Clin. Chim. Acta.

[38] Stingel D., Feldmeier P., Richling E., Kempf M., Elss S., Labib S., Schreier P. (2007). Urinary 2-ethyl-3-oxohexanoic acid as major metabolite of orally administered 2-ethylhexanoic acid in human. Mol. Nutr. Food Res..

[39] Deisinger P.J., Boatman R.J., Guest D. (1994). Metabolism of 2-ethylhexanol administered orally and dermally to the female Fischer 344 rat. Xenobiotica.

[40] Rusyn I., Peters J.M., Cunningham M.L. (2006). Modes of action and species-specific effects of di-(2-ethylhexyl)phthalate in the liver. Crit. Rev. Toxicol..

[41] Reddy J.K., Mannaerts G.P. (1994). Peroxisomal lipid metabolism. Annu. Rev. Nutr..

[42] David R.M., Moore M.R., Cifone M.A., Finney D.C., Guest D. (1999). Chronic peroxisome proliferation and hepatomegaly associated with the hepatocellular tumorigenesis of di(2-ethylhexyl)phthalate and the effects of recovery. Toxicol. Sci. Off. J. Soc. Toxicol..

[43] English J.C., Deisinger P.J., Guest D. (1998). Metabolism of 2-ethylhexanoic acid administered orally or dermally to the female Fischer 344 rat. Xenobiotica.

[44] Bentley P., Calder I., Elcombe C., Grasso P., Stringer D., Wiegand H.J. (1993). Hepatic peroxisome proliferation in rodents and its significance for humans. Food Chem. Toxicol..

[45] Sui H.X., Zhang L., Wu P.G., Song Y., Yong L., Yang D.J., Jiang D.G., Liu Z.P. (2014). Concentration of di(2-ethylhexyl) phthalate (DEHP) in foods and its dietary exposure in China. Int. J. Hyg. Environ. Health.

[46] Heinemeyer G., Sommerfeld C., Springer A., Heiland A., Lindtner O., Greiner M., Heuer T., Krems C., Conrad A. (2013). Estimation of dietary intake of bis(2-ethylhexyl)phthalate (DEHP) by consumption of food in the German population. Int. J. Hyg. Environ. Health.

[47] CPSC United States Consumer Product Safety Commission: Toxicity Review of Di(2-ethylhexyl) Phthalate (DEHP). https://www.cpsc.gov/s3fs-public/ToxicityReviewOfDEHP.pdf.

[48] CSTEE Committee on Toxicity, Ecotoxicity and the Environment: Opinion on Phthalate Migration from Soft PVC Toys and Child-Care Articles. https://ec.europa.eu/health/scientific_committees/environmental_risks/opinions/sctee/sct_out19_en.htm.

[49] Rock C.O., Calder R.B., Karim M.A., Jackowski S. (2000). Pantothenate kinase regulation of the intracellular concentration of coenzyme A. J. Biol. Chem..

[50] Ramaswamy G., Karim M.A., Murti K.G., Jackowski S. (2004). PPARalpha controls the intracellular coenzyme A concentration via regulation of PANK1alpha gene expression. J. Lipid Res..

[51] Skrede S., Halvorsen O. (1979). Increased biosynthesis of CoA in the liver of rats treated with clofibrate. Eur. J. Biochem..

[52] Reilly S.J., Tillander V., Ofman R., Alexson S.E., Hunt M.C. (2008). The nudix hydrolase 7 is an Acyl-CoA diphosphatase involved in regulating peroxisomal coenzyme A homeostasis. J. Biochem..

[53] Theodoulou F.L., Sibon O.C., Jackowski S., Gout I. (2014). Coenzyme A and its derivatives: Renaissance of a textbook classic. Biochem. Soc. Trans..

[54] Schalkwijk J., Jansen P. (2014). Chemical biology tools to study pantetheinases of the vanin family. Biochem. Soc. Trans..

[55] Leighton F., Bergseth S., Rørtveit T., Christiansen E.N., Bremer J. (1989). Free acetate production by rat hepatocytes during peroxisomal fatty acid and dicarboxylic acid oxidation. J. Biol. Chem..

[56] Himms-Hagen J., Harper M.E. (2001). Physiological role of UCP3 may be export of fatty acids from mitochondria when fatty acid oxidation predominates: An hypothesis. Exp. Biol. Med..

[57] Foster D.W. (2012). Malonyl-CoA: The regulator of fatty acid synthesis and oxidation. J. Clin. Investig..

[58] McGarry J.D., Mannaerts G.P., Foster D.W. (1977). A possible role for malonyl-CoA in the regulation of hepatic fatty acid oxidation and ketogenesis. J. Clin. Investig..

[59] Ussher J.R., Lopaschuk G.D. (2008). The malonyl CoA axis as a potential target for treating ischaemic heart disease. Cardiovasc. Res..

[60] Zammit V.A. (1983). Regulation of hepatic fatty acid oxidation and ketogenesis. Proc. Nutr. Soc..

[61] Jenkins B., West J.A., Koulman A. (2015). A review of odd-chain fatty acid metabolism and the role of pentadecanoic Acid (c15:0) and heptadecanoic Acid (c17:0) in health and disease. Molecules.

[62] Mazumder R., Sasakawa T., Ochoa S. (1963). Metabolism of propionic acid in animal tissues. X. Methylmalonyl co-enzyme A mutase holoenzyme. J. Biol. Chem..

[63] Kasumov T., Martini W.Z., Reszko A.E., Bian F., Pierce B.A., David F., Roe C.R., Brunengraber H. (2002). Assay of the concentration and (13)C isotopic enrichment of propionyl-CoA, methylmalonyl-CoA, and succinyl-CoA by gas chromatography-mass spectrometry. Anal. Biochem..

[64] Kasumov T., Cendrowski A.V., David F., Jobbins K.A., Anderson V.E., Brunengraber H. (2007). Mass isotopomer study of anaplerosis from propionate in the perfused rat heart. Arch. Biochem. Biophys..

[65] Badr M.Z., Handler J.A., Whittaker M., Kauffman F.C., Thurman R.G. (1990). Interactions between plasticizers and fatty acid metabolism in the perfused rat liver and in vivo. Inhibition of ketogenesis by 2-ethylhexanol. Biochem. Pharmacol..

[66] Manninen A., Kroger S., Liesivuori J., Savolainen H. (1989). 2-Ethylhexanoic acid inhibits urea synthesis and stimulates carnitine acetyltransferase activity in rat liver mitochondria. Arch. Toxicol..

[67] Olowe Y., Schulz H. (1980). Regulation of thiolases from pig heart. Control of fatty acid oxidation in heart. Eur. J. Biochem..

[68] Dalluge J.J., Gort S., Hobson R., Selifonova O., Amore F., Gokarn R. (2002). Separation and identification of organic acid-coenzyme A thioesters using liquid chromatography/electrospray ionization-mass spectrometry. Anal. Bioanal. Chem..

[69] Zhang G.F., Kombu R.S., Kasumov T., Han Y., Sadhukhan S., Zhang J., Sayre L.M., Ray D., Gibson K.M., Anderson V.A. (2009). Catabolism of 4-hydroxyacids and 4-hydroxynonenal via 4-hydroxy-4-phosphoacyl-CoAs. J. Biol. Chem..

[70] Li Q., Zhang S., Berthiaume J.M., Simons B., Zhang G.F. (2014). Novel approach in LC-MS/MS using MRM to generate a full profile of acyl-CoAs: Discovery of acyl-dephospho-CoAs. J. Lipid Res..

[71] Corkey B.E., Hale D.E., Glennon M.C., Kelley R.I., Coates P.M., Kilpatrick L., Stanley C.A. (1988). Relationship between unusual hepatic acyl coenzyme A profiles and the pathogenesis of Reye syndrome. J. Clin. Investig..

[72] Grosse S.D., Khoury M.J., Greene C.L., Crider K.S., Pollitt R.J. (2006). The epidemiology of medium chain acyl-CoA dehydrogenase deficiency: An update. Genet. Med..

[73] Mitchell G.A., Gauthier N., Lesimple A., Wang S.P., Mamer O., Qureshi I. (2008). Hereditary and acquired diseases of acyl-coenzyme A metabolism. Mol. Genet. Metab..

[74] Dansie L.E., Reeves S., Miller K., Zano S.P., Frank M., Pate C., Wang J., Jackowski S. (2014). Physiological roles of the pantothenate kinases. Biochem.Soc. Trans..

